# Subduction-related oxidation of the sublithospheric mantle evidenced by ferropericlase and magnesiowüstite diamond inclusions

**DOI:** 10.1038/s41467-022-35110-x

**Published:** 2022-12-06

**Authors:** Ekaterina S. Kiseeva, Nester Korolev, Iuliia Koemets, Dmitry A. Zedgenizov, Richard Unitt, Catherine McCammon, Alena Aslandukova, Saiana Khandarkhaeva, Timofey Fedotenko, Konstantin Glazyrin, Dimitrios Bessas, Georgios Aprilis, Alexandr I. Chumakov, Hiroyuki Kagi, Leonid Dubrovinsky

**Affiliations:** 1 School of Biological, Earth and Environmental Sciences, University College Cork, Cork, Ireland; 2 Institute of Precambrian Geology and Geochronology of the Russian Academy of Sciences, nab. Makarova 2, St. Petersburg, 199034 Russia; 3 Bayerisches Geoinstitut, Universität Bayreuth, D-95440 Bayreuth, Germany; 4 A.N. Zavaritsky Institute of Geology and Geochemistry, 15 Vonsovskogo street, Ekaterinburg, 620016 Russia; 5 Ural State Mining University, 30 Kuibysheva street, Ekaterinburg, 620014 Russia; 6 Materials Physics and Technology at Extreme Conditions, Laboratory of Crystallography, Universität Bayreuth, D-95440 Bayreuth, Germany; 7 Deutsches Elektronen-Synchrotron DESY, Notkestr. 85, 22607 Hamburg, Germany; 8 ESRF-The European Synchrotron, CS 40220, 38043 Grenoble, Cedex 9 France; 9 Geochemical Research Center, Graduate School of Science, The University of Tokyo, Tokyo, 113-0033 Japan

**Keywords:** Mineralogy, Petrology, Geochemistry, Geology

## Abstract

Ferropericlase (Mg,Fe)O is the second most abundant mineral in Earth’s lower mantle and a common inclusion found in subcratonic diamonds. Pyrolitic mantle has Mg# (100 × Mg/(Mg+Fe)) ~89. However, ferropericlase inclusions in diamonds show a broad range of Mg# between 12 and 93. Here we use Synchrotron Mössbauer Source (SMS) spectroscopy and single-crystal X-ray diffraction to determine the iron oxidation state and structure of two magnesiowüstite and three ferropericlase inclusions in diamonds from São Luiz, Brazil. Inclusion Mg#s vary between 16.1 and 84.5. Ferropericlase inclusions contain no ferric iron within the detection limit of SMS, while both magnesiowüstite inclusions show the presence of monocrystalline magnesioferrite ((Mg,Fe)Fe^3+^_2_O_4_) with an estimated 47–53 wt% Fe_2_O_3_. We argue that the wide range of Fe concentrations observed in (Mg,Fe)O inclusions in diamonds and the appearance of magnesioferrite result from oxidation of ferropericlase triggered by the introduction of subducted material into sublithospheric mantle.

## Abstract (web summary)

This article reports finding of a highly oxidised mineral in diamond inclusion derived from mantle transition zone or lower mantle, very reduced areas on our planet. Such oxidised material is likely linked to subduction of carbonates into this region.

## Introduction

**Fig. 1:**
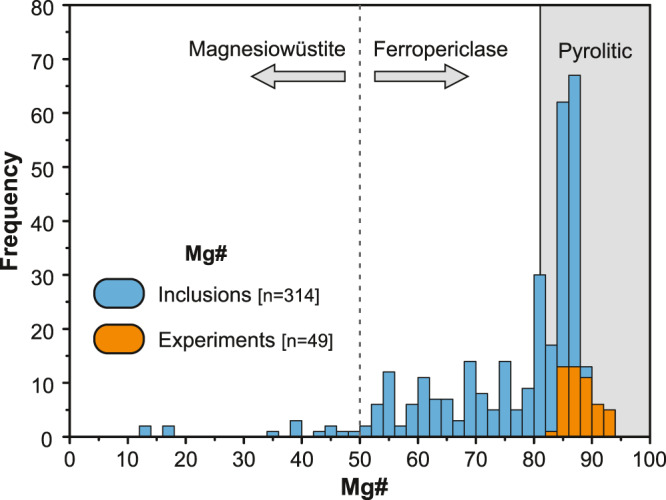
Mg# of (Fe,Mg)O inclusions in diamonds from localities worldwide and selected experimental studies in pyrolite and fertile lherzolite KLB-1 compositions. References to the literature data are listed in Supplementary Material.

The lower mantle comprises >50% of Earth’s volume, and compositionally is considered largely homogeneous and primitive or pyrolitic^1,2^. It has been acknowledged, however, that modern-day subducted slabs can penetrate deep into the lower mantle, causing heterogeneities and locally oxidised regions (e.g. ref. 3). The mineralogy of the upper part of the lower mantle is relatively simple: in a pyrolitic system it should consist of ~70 vol% bridgmanite ((Mg, Fe)SiO_3_), <20 vol% ferropericlase (Mg,Fe)O and <10 vol% Ca-Si-perovskite (CaSiO_3_)^4,5^. Diamonds and their inclusions are the only available natural samples from Earth’s lower mantle. Of the more than 650 sublithospheric inclusions reported to date ferropericlase is the most abundant^6^, comprising some 40% of the population. These are commonly assumed to form in the lower mantle^7,8^. Figure 1 shows the distribution of Mg# in magnesiowüstite (Mg# < 50) and ferropericlase (Mg# > 50) inclusions in lower mantle diamonds reported in the literature.

The wide range of compositions displayed in Fig. 1 and the extreme Fe-enrichment (up to 93 wt% FeO) is unlikely to have resulted from a single mechanism. As a consequence, only a fraction of the reported inclusions could be in equilibrium with bridgmanite in pyrolitic lower mantle or with garnet and ringwoodite at the mantle transition zone, suggesting that the sublithospheric mantle is likely to contain highly heterogeneous regions with non-pyrolitic compositions. These regions may contain transported sediments and oceanic crust from the surface of subducting slabs, which are also likely to be more oxidised than the ambient sublithospheric mantle, e.g., ref. 9.

The purpose of this study is to measure the oxidation state of iron in ferropericlase and magnesiowüstite inclusions in diamond displaying a range of Mg# and to explore the link between their compositions and iron oxidation state. This has particular importance for the storage of oxidised material in the deep mantle, as well as for the speciation of deep mantle fluids, diamond formation, rheological, and melting properties at the depths inaccessible for direct sampling.

## Results

Five diamonds, 4–5 mm in size, recovered from alluvial deposits in Sao Luiz, Juina, Brazil were selected for this study. The diamonds were polished flat on both sides so that the inclusions were exposed to the surface prior to analysis. Their size ranged between 20 and 80 μm. Major element compositions of the inclusions are listed in Supplementary Table 1 and were previously reported by Zedgenizov et al. (refs. 10, 11). Three inclusions (SL14, SL14_2 and SL24) are ferropericlase with Mg# = 79–85, and two inclusions (SL82 and SL5_2) are magnesiowüstite with Mg# = 16 and 40, respectively. Their minor element concentrations vary within 0.06–1.25 wt% Cr_2_O_3_, 0.17–1.68 wt% MnO and 0.06–1.19 wt% NiO.

In order to determine the crystal structure and oxidation state of Fe in the studied inclusions, we used single crystal X-ray diffraction analysis (beamline P02.2, PETRA III, DESY,  with beam size ~2 × 2 µm^2^ at FWHM) combined with Synchrotron Mössbauer Source (SMS) spectroscopy (the Nuclear Resonance beamline^12^ ID18 at the European Synchrotron Radiation Facility, Grenoble, with beam size 3 × 9 µm^2^ at FWHM). All inclusions were initially studied by X-ray diffraction. Mg-rich inclusions SL14, SL14_2, and SL24 contained monophase ferropericlase single crystals (Supplementary Table 2). The ferric iron content of these inclusions, analysed by Mössbauer spectroscopy, was below the detection limit of ~0.03 Fe^3+^/Fe_tot_ (Supplementary Table 3, Supplementary Figs. 1–3).

**Fig. 2:**
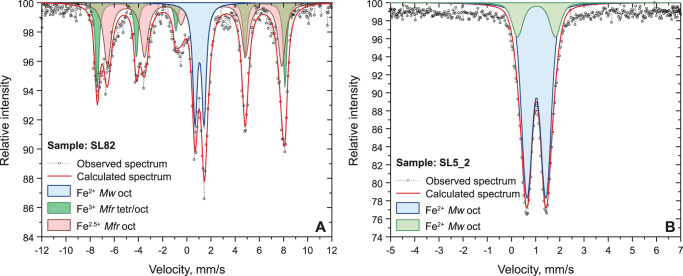
Mössbauer spectra of inclusions SL82 (A) and SL5_2 (B). **A** Blue doublet corresponds to Fe^2+^ in the octahedral site of magnesiowüstite. Green magnetic sextet corresponds to Fe^3+^ in octahedral and tetrahedral sites of magnesioferrite, red sextet corresponds to Fe^2.5+^; i.e., Fe^3+^↔Fe^2+^ rapid electron hopping between octahedral sites of magnesioferrite. **B** Green and blue doublets correspond to Fe^2+^ in the octahedral site of magnesiowüstite.

Single crystal X-Ray diffraction identified single crystal inclusions with the sizes larger than 2–5 μm based on X-ray absorption on inclusions exposed at the diamond surface and inside the diamond. X-ray diffraction of SL82 and SL5_2 confirmed the presence of two coexisting monocrystalline phases, magnesiowüstite and magnesioferrite. X-ray absorption was used in order to locate and centre on X-ray beam inclusions and the sizes were determined from absorption scans. These scans show different phases spatially separated (i.e. that is not intergrowth) but crystallographically orientated ([111] direction of spinel-structured phase parallel to the [100] direction of the cubic phase). This relationship indicates that magnesiowüstite and magnesioferrite likely unmixed from a different precursor phase and either crystallised together and were trapped as a composite inclusion or magnesioferrite exsolved from magnesiowüstite after entrapment. Based on XRD and SMS data, magnesioferrite has a magnetite structure or inverse spinel, with some divalent iron substituted by magnesium. Indirect estimates from the integrated peak areas of Mössbauer spectra for SL82 sample (Fig. 2A) are in a good agreement with X-ray diffraction data, identifying two phases containing iron. The signal for SL5_2, however, is too low to resolve for the ferric iron doublet and the fit of SL5_2 shows only magnesiowüstite (Fig. 2B). Relative areas in the Mössbauer spectrum (Fig. 2A) combined with chemical compositions allow us to estimate the proportion of magnesiowüstite in the SL82 inclusion as 42% (considering only the molar ratio of iron-bearing phases, which are magnesiowüstite and magnesioferrite 42% and 58%, respectively). Nevertheless, scanning electron microscopy showed no magnesioferrite either as single monocrystalline inclusions or as multiple exsolution phases on the surface of inclusion SL82 (Supplementary Fig. 4). Thus, we infer magnesioferrite in SL82 and SL5_2 to be located under the surface. The compositions of magnesioferrite calculated from the X-ray diffraction and SMS data are reported in Supplementary Table 4.

Magnesioferrite has previously been reported with magnesiowüstite, but mainly as small, nanometre-size crystals in magnesiowüstite/ferropericlase matrix, ascribed to either exsolution in ferropericlase^7,13,14^ or the breakdown of complex iron oxides^15,16^. Interestingly, all magnesioferrite inclusions in diamonds reported in the literature, including the results of this study, coexist either with Fe-rich ferropericlase or, more often, with magnesiowüstite.

### Ferric iron concentration in ferropericlase

The concentration of Fe^3+^ in ferropericlase has been addressed by a number of experimental studies that generally agree on very low solubility at lower mantle pressures (usually below 10% ferric iron)^17–20^ and high solubility, up to 70%, at upper mantle conditions of <5 GPa^21^.

In order to investigate the effect of thermodynamic parameters, Otsuka et al.^22^ studied the solubility of Fe^3+^ in (MgFe)O, as a function of pressure (P), temperature (T), oxygen fugacity (*f*O_2_) and mineral composition. The authors concluded that the solubility strongly decreases with increasing pressure and decreasing *f*O_2_^22,23^ and strongly increases with increasing wüstite component. If applied to the compositions of SL82 and SL5_2, according to this parameterisation, magnesiowüstite with 100*X_Fe_ = 60 that corresponds to the SL5_2 inclusion, is estimated to have around 0.03 Fe^3+^/Fe_tot_ at IW buffer and 0.11 Fe^3+^/Fe_tot_ at Ni-NiO buffer at 24 GPa and 1873 K. At 15 GPa at the same temperature this ratio is significantly higher, with ~0.08 Fe^3+^/Fe_tot_ at IW and 0.20 Fe^3+^/Fe_tot_ at Ni-NiO. The composition of 100*X_Fe_ = 83, that corresponds to the SL82 inclusion, is beyond the reported interval of compositions investigated by Otsuka et al.^22^ and therefore subject to large uncertainties. Extrapolation of the parameterisation to this composition yields 0.055 Fe^3+^ per 1 oxygen formula unit at IW and 0.20 Fe^3+^/Fe_tot_ at Ni-NiO at 24 GPa and 1873 K, which translates into 0.07 and 0.27 Fe^3+^/Fe_tot_ at IW and Ni-NiO, respectively. At 15 GPa and 1873 K the model yields 0.18 Fe^3+^/Fe_tot_ at IW and 0.6 Fe^3+^/Fe_tot_ at Ni-NiO, respectively. These values agree with Frost and Langenhorst^24^, who ran experiments in more Fe-rich compositions and reported up to 0.19 Fe^3+^/Fe_tot_ in magnesiowüstite with 100*X_Fe_ = 55 at 25 GPa and 1650 ^o^C, which broadly supports the model proposed by ref. 22.

Despite seemingly large uncertainties to the model and possible overestimation of Fe^3+^ contents at wüstite-rich compositions, it appears reasonable to assume that in relatively oxidised areas of the sublithospheric mantle, (Mg,Fe)O and in particular its wüstite-rich varieties can contain significant amounts of ferric iron.

### Source lithology for ferropericlase-magnesiowüstite inclusions

Among the inclusions that we studied, only ferropericlase from SL24, SL14 and SL14_2 can theoretically be in equilibrium with bridgmanite in a pyrolitic mantle^11^. This allows us to calculate the #Mg of the system as 90–92, consistent with a pyrolitic (or similar) bulk rock lower mantle source. Although there are few bridgmanite-ferropericlase pairs reported in the literature, using experimental data it is possible to estimate the approximate Mg# of the system and relate it to the potential lithology from which (Mg,Fe)O crystallised.

Experimental work on stability field of the lower mantle phases and their composition can be subdivided into Al-free and Al-bearing systems. In Al-free systems, close to harzburgitic, ferropericlase will have a lower Mg# than bridgmanite^6,25,26^.

For pressures at the top of the lower mantle (25–30 GPa), the distribution coefficient (K) between ferropericlase (Fp) and bridgmanite (Bdm), ${K}^{{Fp}-{Bdm}}={\left(\frac{{X}_{{Fe}}}{{X}_{{Mg}}}\right)}^{{Fp}}/{\left(\frac{{X}_{{Fe}}}{{X}_{{Mg}}}\right)}^{{Bdm}}$ranges between 2 and 8 in Al-free systems and decreases with pressure to <2 at 60 GPa and greater^27^. This is in good agreement with ${K}^{{Fp}-{Bdm}}$ measured for enstatite-ferropericlase inclusions found in the same diamonds (between ~3 and 10)^28,29^.

Thus, in support of the data shown in Fig. 1, even by the most conservative estimates, only ~50% of all ferropericlase inclusions reported in the literature could potentially be derived from pyrolitic mantle through the breakdown of γ-spinel or majoritic garnet, and all other ferropericlase inclusions must have crystallised through a different mechanism, explored in more detail below.

### Isotopic signatures of diamonds hosting ferropericlase inclusions

**Fig. 3:**
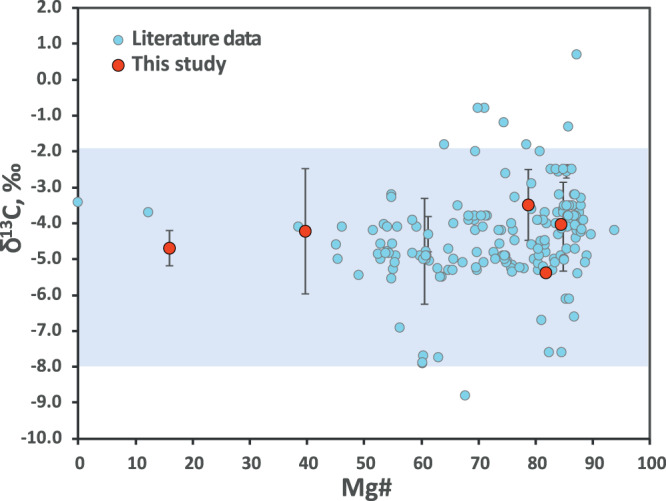
Carbon isotopic composition of diamonds hosting ferropericlase and magnesiowüstite inclusions. In light blue is shown the mantle range^30^. References to literature data are listed in Supplementary Material.

Carbon isotopic composition has been shown to be an excellent indicator of diamond protolith^30,31^. For lithospheric inclusions in diamonds, ~90% of peridotitic diamonds fall into the so-called mantle interval of −5‰ ± 3‰ (ref. 32), while eclogitic diamonds show a skewed distribution to much lighter values, indicating the potential source of eclogitic carbon, which derives from the organic carbon subsequently oxidised to carbonate in the deeper portions of oceanic crust^31,33^. Studies exploring carbon isotopic signatures of sublithospheric diamonds^11,34–37^ report a large variation in isotopic compositions depending on the type of inclusion and locality^6^. Diamonds encapsulating majoritic garnets and Ca-perovskite inclusions show a wide range of δ^13^C between 5‰ and −25‰, with most compositions being significantly lighter than the accepted mantle range^30,34^, inferring their potential link to organic carbon. Unlike other superdeep diamonds, ferropericlase-bearing diamonds show a much more limited carbon isotope range which correspond with typical mantle values (Fig. 3).

This distribution of values may be correlated with their geographic location. In the case of majoritic inclusions in diamonds, the carbon isotopic signature of their hosting diamond is strongly related to the craton from which they are derived. South African diamonds hosting majoritic garnets have significantly lower carbon isotopic values than West African and Brazilian diamonds^30^. Carbon isotopic data for diamonds hosting ferropericlase inclusions are limited to mainly three cratons: Amazonian, Slave, and West African (Fig. 3), and unlike the case of majoritic garnets, the majority of diamonds, regardless of craton, are clustered around the mantle carbon value. This may suggest that the mantle portion of the slab is involved in ferropericlase-bearing diamond formation.

Despite a mantle-like isotopic signature, a subduction source of carbon cannot be ruled out^33,37^, especially in the light of data from other diamonds from the same craton linked to surface origins^34^. The carbon isotopic signatures of altered oceanic crust span a wide range of δ^13^C = ~3‰ and δ^13^C = ~−35‰ (refs. 37, 38). This range covers the mantle values making it difficult to place a definite constraint on the source of carbon. However, the carbon isotope difference between diamonds encapsulating ferropericlase and other types of inclusions raises a number of questions.

One hypothesis generated from this study is linked to the depth associated with ferropericlase inclusions. It is possible that the Amazonian subducted slab had already lost most of its shallow carbon by the time it reached the transition zone and the lower mantle pressures; the source of carbon released at those depths is associated with the mantle portion of the slab. This is consistent with the deep release of water from this part of the slab expected in the lower transition zone and shallow upper mantle^39,40^. These fluids could then dissolve mantle-like carbon from the subducted lithospheric mantle and react with iron-rich melts that exist within the slab itself, contributing to diamond formation in the vicinity of iron-rich melts or iron metal^41,42^. This scenario is also supported by the fact that deeper inclusions seem more isotopically uniform than shallower inclusions^33,37^, although sampling bias and other limitations should also be taken into account.

A second hypothesis describes a mixing line between initial mantle-derived carbon and carbon introduced by a subducting slab. As shown in Fig. 3, ferropericlase of pyrolitic composition shows larger variations in diamond-host carbon isotopic composition compared to non-pyrolitic ferropericlase. If we assume that initial lower mantle ferropericlase was slightly more Fe-rich, consistent with Fe-rich non-pyrolitic lower mantle, then by reacting with subduction-introduced MgCO_3_-rich fluids or melts, ferropericlase could increase its Mg# while diamond would be characterised by larger variations in carbon isotopic compositions depending on the type of subducted carbonate.

### Depth and mechanism of formation of ferropericlase inclusions in diamonds

The depth of formation of ferropericlase inclusions in diamonds is still a matter of debate. In peridotitic compositions, the stability field of (Mg,Fe)O starts at the base of the mantle transition zone and extends throughout the entire lower mantle^43^, which supports the assumption of an ultra-deep origin for the studied inclusions. In addition, the lower mantle origin of some ferropericlase inclusions can be indirectly evidenced by the presence of lower mantle minerals encapsulated by the same diamond, such as bridgmanite and Ca-Si-perovskite^10,20,39^. This method is not infallible, however, as non-touching mineral inclusions, although present within the same diamond could have been captured at different depths. In addition, the included minerals frequently do not retain their high-pressure structures. Of the studied diamonds and their inclusions only the diamond-host of SL14_2 contained another inclusion—bridgmanite (enstatite inferred to be former bridgmanite)^11^, with ferropericlase and bridgmanite inclusions being non-touching.

Although plausible, the base of the transition zone and lower mantle, however, are not the only possible regions of formation of ferropericlase in the mantle. A large number of experimental studies have argued in favour of ferropericlase crystallisation at shallower depths^44–48^. These studies report crystallisation of ferropericlase under upper mantle conditions (0.5–12 GPa) in association with olivine in harzburgitic carbonate-rich silica-undersaturated compositions^45,48^ or within an assemblage of iron-rich carbonated peridotite^44^.

Thus, determining the depth of ferropericlase inclusion formation is relatively complex. However, some assumptions about the minimum formation depth, especially of single inclusions, can still be made based on their ferric iron content. Although the solubility of ferric iron is substantially lower in periclase-rich compositions, according to the model by Otsuka et al.^22^ at depths <5 GPa and relatively high oxygen fugacities, it would still be sufficiently high to be detected by Mössbauer spectroscopy, especially those collected using the SMS that has a detection limit of ~0.03 Fe^3+^/Fe_tot_. At pressures above 5 GPa at IW buffer the solubility of ferric iron in ferropericlase will be below 0.01 (ref. 22). Higher oxidation states will shift the lower limit of pressures to higher values, because ferric iron content increases with increasing *f*O_2_, but even at Re-ReO_2_ buffer at pressures of 15–24 GPa the solubility of ferric iron in ferropericlase with X_Fe_ = 0.2 will be below 0.03–0.04. In all three inclusions (SL14, SL14_2 and SL24) the ferric iron content is below the detection limit of the SMS, implying pressures >4–5 GPa (depending on composition and *f*O_2_) at all buffers. The high solubility of ferric iron in magnesiowüstite precludes the formation of SL5_2 and SL82 at pressures below 5 GPa.

Our results agree with previous findings that (Mg,Fe)O does not incorporate a large amount of ferric iron at high pressure^17,20,22^. X-ray diffraction analysis confirms that ferric iron in the system is partitioned into magnesioferrite. Based on X-ray diffraction data, we calculate iron-magnesium partition coefficients ${K}^{{Mw}-{Mfr}}={\left(\frac{{{X}_{{Fe}}}^{2+}}{{X}_{{Mg}}}\right)}^{{Mw}}/{\left(\frac{{{X}_{{Fe}}}^{2+}}{{X}_{{Mg}}}\right)}^{{Mfr}}$for magnesiowüstite (Mw) and magnesioferrite (Mfr) as 5.6 and 7.2 for SL5_2 and SL82, respectively. These values are in reasonable agreement with values for magnesiowüstite—(Mg,Fe)SiO_4_ (spinel) at 17–21 GPa^49^, supporting formation of the studied inclusions at the mantle transition zone or uppermost parts of the lower mantle.

In relation to the formation mechanism of wüstite-rich varieties that span a wide range of compositions (Fig. 1), there are several scenarios proposed in the literature. They include: (1) interaction of carbonated melts with iron melt-bearing peridotite at a wide range of sublithospheric upper mantle and transition zone pressures^44^; (2) formation at the core-mantle boundary or at significant depths (>1700 km)^13,28,50^; (3) continuous decarbonation reaction with increasing pressure in the lower mantle^51^; (4) oxidation of pyrrhotite with diamond precipitation^52^; (5) breakdown of complex Fe-oxides^15,53,54^; as well as (6) mantle metasomatism and fractional crystallisation processes.

Despite the challenge to provide a definite answer to the exact mechanism of formation, since carbon is present in the system, it is likely that redox reactions involving either subducted solid carbonate, carbonate melt or carbonated fluid have occurred. This is also supported by an experimental study that showed the coexistence of magnesiowüstite and magnesioferrite at lower mantle conditions in the presence of carbonate^55^, while no magnesiowüstite + magnesioferrite stability field was observed in carbonate-free systems^53,54^. The wide range of intermediate compositions between the wüstite and periclase endmembers observed as inclusions in diamonds and the presence of magnesioferrite associated with wüstite-rich varieties can be explained by continuous oxidation of iron metal, or iron-rich melt with the formation of ferropericlase/magnesiowüstite compositions, depending on the Fe-budget of the initial lithology, in agreement with experimental works that produced a wide range of ferropericlase compositions with respect to Mg#, Na and Ni that closely mimics the inclusion range except for the most rare wüstite-rich composition (Fig. 1)^44,46,48^.

**Fig. 4:**
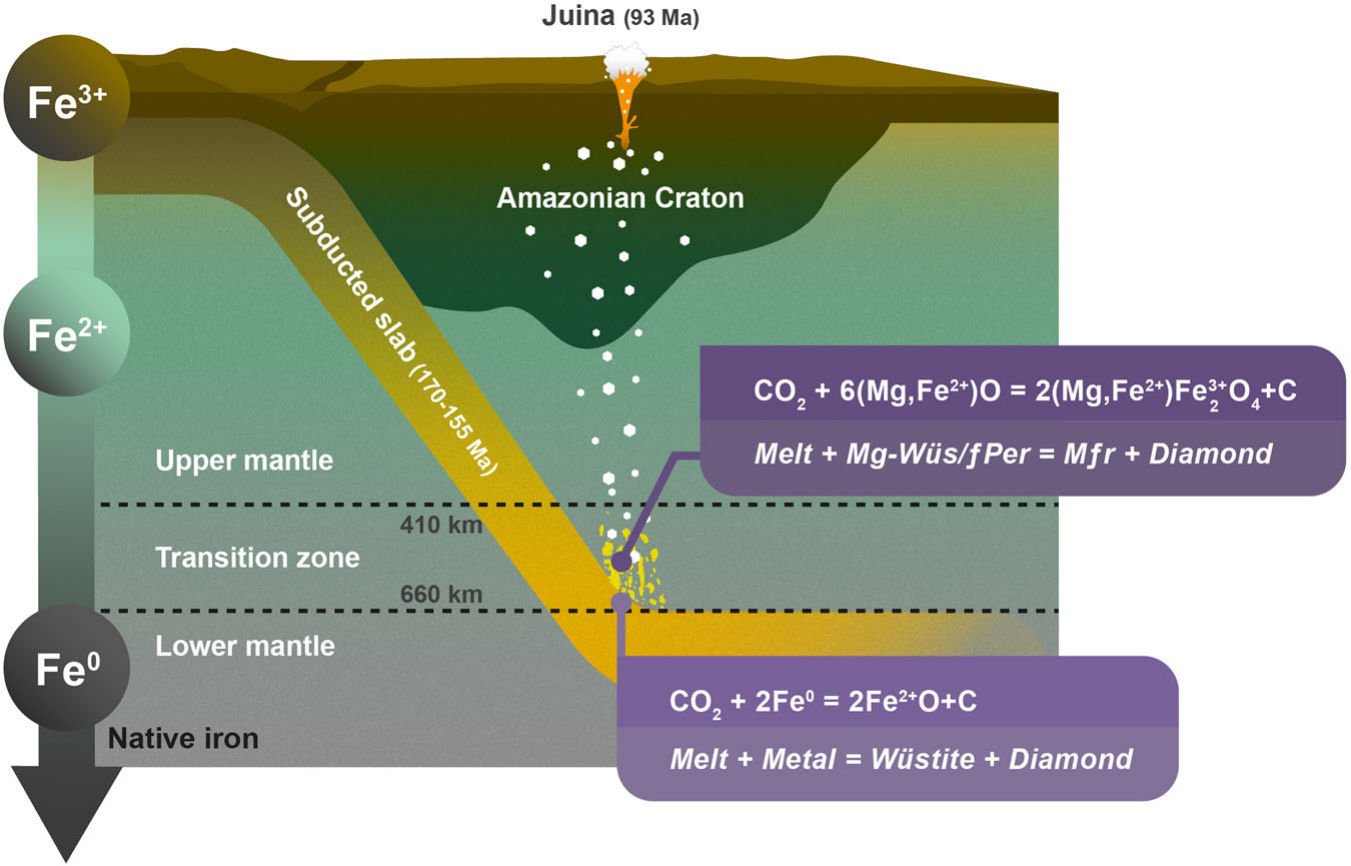
Mantle beneath the Amazonian Craton. Schematic illustration showing the formation of the magnesiowüstite-magnesioferrite mineral association in Juina diamonds.

In order to also crystallise magnesioferrite, we suggest a two-stage process in addition to the proposed mechanism for magnesiowüstite formation that involves gradual oxidation of a metal-bearing mantle assemblage by carbonated fluids transported by subduction (Fig. 4). During the first stage of oxidation, carbonated fluids or melts react with Fe metal following the reaction:

1 $$\begin{array}{ccc}{{{{{{\rm{CO}}}}}}}_{2}+2{{{{{{\rm{Fe}}}}}}}^{0} &=& 2{{{{{{\rm{Fe}}}}}}}^{2+}{{{{{\rm{O}}}}}}+{{{{{\rm{C}}}}}}\\ {Melt}+{Metal} &=& W{{ \ddot{{u}} }}{stite}+{Diamond}\end{array}$$

The likely presence of Mg in carbonated melt, if the reaction proceeds to the right, will lead to the formation of magnesiowüstite. Reaction (1) will take place until Fe metal is fully exhausted, at which point the *f*O_2_ of the system can shift to above the iron-wüstite buffer (IW). With ongoing reaction, carbonated melt oxidises the produced magnesiowüstite and already existing ferropericlase with formation of magnesioferrite as follows:

2 $$	{{{{{{\rm{CO}}}}}}}_{2}+6({{{{{\rm{Mg}}}}}},{{{{{{\rm{Fe}}}}}}}^{2+}){{{{{\rm{O}}}}}}=2({{{{{\rm{Mg}}}}}},{{{{{{\rm{Fe}}}}}}}^{2+}){{{{{{\rm{Fe}}}}}}}_{2}^{3+}{{{{{{\rm{O}}}}}}}_{4}+{{{{{\rm{C}}}}}} \\ 	\quad{Melt}+{magnesiow}{{ \ddot{u} }}{stite}/{ferropericlase}={magnesioferrite}+{Diamond}$$

A similar process was proposed for magnesioferrite coexisting with ankerite found in Xiuyan crater, China, where the peak shock pressure was estimated to be 35–45 GPa (ref. 56). Following reaction (1), the oxidation of metallic iron containing ~10 wt% Ni and 1 wt% S (ref. 57) could result in the formation of sulphide, the most common inclusion in diamond^58^.

The source of carbonate for reactions (1) and (2) could be either carbonated fluid released from the slab upon melting of carbonated eclogites or peridotites^40,59,60^, or, for example, slab regions enriched in sedimentary carbonate or a mixture between mantle carbon and sedimentary carbonate^44^. Wüstite-rich compositions could have also derived from interaction with iron-rich carbonates. It was recently shown that Fe-rich carbonates could survive subduction to depths of the lower mantle^61^.

### Oxidised regions of Earth’s sublithospheric mantle

The mantle transition zone and the lower mantle are thought to be significantly more reduced than the upper mantle^57^. With increasing pressure, ferric iron becomes more soluble in silicate minerals. As a result, ferrous iron disproportionates into metallic iron and ferric iron^62^, i.e.,

3 $$3{{{{{{\rm{Fe}}}}}}}^{2+}{{{{{\rm{O}}}}}}={{{{{{\rm{Fe}}}}}}}^{0}+{{{{{{\rm{Fe}}}}}}}_{2}^{3+}{{{{{{\rm{O}}}}}}}_{3}$$

This process starts in the lowermost upper mantle and mantle transition zone, increasing the concentration of ferric iron in garnet^9,62^ and continues in the lower mantle as bridgmanite is stabilised. Although not yet documented in natural samples, it has been shown experimentally that ferrous iron in bridgmanite disproportionates with precipitation of metallic iron and stabilisation of the FeAlO_3_ component^1,63^, leading to Fe^3+^/Fe_tot_ ratios in bridgmanite of 0.5–0.6 (refs. 57, 63). If the lower mantle has a similar amount of oxygen as the upper mantle, then its *f*O_2_ should be between 0 and −1.5 relative to the iron-wüstite buffer (IW) (ref. 57).

Although the exact pressure of formation is unknown, the presence of magnesioferrite in equilibrium with magnesiowüstite allows us to estimate the *f*O_2_ of the system following the reaction:

4 $$6({{{{{\rm{Mg}}}}}},{{{{{\rm{Fe}}}}}}){{{{{\rm{O}}}}}}+{{{{{{\rm{O}}}}}}}_{2}=2({{{{{\rm{Mg}}}}}},{{{{{\rm{Fe}}}}}}){{{{{{\rm{Fe}}}}}}}_{2}{{{{{{\rm{O}}}}}}}_{4}$$

Due to uncertainties in *f*O_2_ arising from the mineral compositions, buffer equilibria and the pressure and temperature of formation of inclusions, we estimate *f*O_2_ to be within the range between IW+5 to IW+8. The gradual oxidation of iron-bearing metal through its reaction with subducted carbonate in sublithospheric mantle provides a mechanism for (1) formation of ferropericlase of various compositions including highly Fe-rich varieties^44^, (2) formation of magnesioferrite and (3) formation of diamond hosts for inclusions. Magnesioferrite inclusions in diamonds derived from sublithospheric mantle contain Fe^3+^/Fe_tot_ ratios consistent with an *f*O_2_ significantly above the IW buffer. This suggests that subducted carbonate acting as an oxidising agent, can lead to the formation of oxidised metal-free regions in Earth’s sublithospheric mantle.

## Methods

### Samples

Three ferropericlase and two magnesiowüstite inclusions in diamonds investigated in this study originate from alluvial deposits of the São Luis River (Juina, Brazil) (more details about the host diamonds and inclusion compositions are given in ref. 11, Supplementary Table 5 and Supplementary Fig. 5). The diamonds were polished flat on both sides so that the inclusions were exposed to the surface prior to analysis. Their size ranged between 20 and 80 μm. All measurements were performed on loose diamonds.

### Mössbauer spectroscopy

Mössbauer absorption spectra were collected at ambient temperature at the Nuclear Resonance beamline (ID18) (ref. 12) of the European Synchrotron Radiation Facility (Grenoble, France) using a Synchrotron Mössbauer Source (SMS) (ref. 64). The accelerator was operating in 7/8 + 1 bunch mode with an electron current of 200 mA in top-up mode. The typical X-ray beam size [full width at half maximum (FWHM)] was 3.7(5) × 8.2(5) μm^2^ (H × V). The line width of the SMS was determined before and after collection of each spectrum of the sample by measuring a Mössbauer absorption spectrum by a reference single line absorber, i.e., K_2_Mg^57^Fe(CN)_6_. A typical linewidth of the source was determined to be 12(1) neV. More information about sample mounting and alignment procedure is given in ref. 65. Each sample spectrum was collected for about 4 h over a velocity range of ±6 or ±12 mm/s depending on whether magnetic interactions were present or not. Velocity scales were calibrated using Fe foil.

Mössbauer spectra were fitted to quadrupole doublets and magnetic sextets using MossA software^66^ with a full transmission integral assuming a Lorentzian-squared line shape for the instrumental function. The fitted parameters were centre shift (CS), quadrupole splitting (QS), FWHM of the linewidth, hyperfine magnetic field (B) and area (I). Centre shift values are reported relative to *α*-iron at ambient conditions.

X-ray optical components at ESRF, and particularly at the ID18 beam-line, are carefully selected in order to contain the minimum amount (ppm level) of iron. Generally, this amount of iron does not affect SMS spectra due to the strong signal from the sample. However, due to the low natural abundance of ^57^Fe, i.e., about 2%, the signal from the sample was sufficiently weak that spectral contamination from iron in the X-ray optical components could be detected. In order to account for this effect at each experimental run, i.e., for different combinations of X-ray optical components, SMS spectra were measured without any sample so that Mössbauer absorption due to the optical components could be accurately determined for each of the sample SMS spectra. The Mössbauer absorption solely due to the optical components was found to contribute <0.5% in the measured absorption lines.

### X-ray diffraction

X-ray diffraction (XRD) measurements were performed at the Extreme Conditions Beamline P02.2 at PETRA III (Hamburg, Germany)^67^. Data were acquired with a PerkinElmer XRD1621 flat panel detector, X-ray beam-size 2 × 2 μm^2^ (FWHM), and wavelength *λ* = 0.2885 Å. Inclusions were brought to the centre of rotation of goniometer (w-angle) in two steps: coarse tuning of a sample position by the on-line microscope setup, which was aligned with respect to the X-ray beam, and fine-tuning of the sample position by employing standard X-ray absorption cantering procedure. XRD wide-scan images were collected during continuous rotation of the samples from –20 to +20° on the omega axis; single crystal data collection experiments were performed by narrow 0.5° scanning *ω*-scanning in the range from −35° to +35°.

Data integration and absorption corrections were performed with CrysAlisPro software (ref. 68) version 171.38.43. Refinement was performed using the JANA2006 (ref. 69) version from 25.10.2015.

### XRD data analysis

Analysis of collected diffraction images and cell refinement with further data reduction was performed using CrysAlis^Pro^ software^68^. Ewald^Pro^ reciprocal space observation tool implemented in CrysAlis^Pro^ allowed separation and independent treatment of individual single-crystal domains within the analysed spots in such cases when the sample was multi-crystalline. For each single crystal domain this tool permitted to find its independent orientation matrix, to define the unit cell parameters, and to subsequently extract Bragg peak intensities. Structure solution and refinement was performed using JANA2006 (ref. 69).

### Scanning electron microscopy

Scanning electron microscope JEOL JMS-6360 was used for backscattered electron image acquisitions at Bayreuth Geoinstitute. Images were obtained using acceleration voltage of 15 kV and probe current of 0.43 nm.

## Supplementary information

Supplementary Information

## Data Availability

The authors declare that the data are provided in the article and in the supplementary material.
