# Supplementary material for: Subduction-related oxidation of the sublithospheric mantle evidenced by ferropericlase and magnesiowüstite diamond inclusions
Source: Nat Commun. 2022 Dec 6;13:7517. doi: 10.1038/s41467-022-35110-x (PMC9726884; doi:10.1038/s41467-022-35110-x)
Supplement: Supplementary file 1 — Supplementary Information [file 41467_2022_35110_MOESM1_ESM.pdf]

Supplementary Information
for

Subduction-related oxidation of the sublithospheric mantle evidenced by ferropericlase and magnesiowüstite diamond inclusions

Ekaterina S. Kiseeva[1,*], Nester Korolev[2], Iuliia Koemets[3], Dmitry A. Zedgenizov[4,5], Richard Unitt[1], Catherine McCammon[3], Alena Aslandukova[3], Saiana Khandarkhaeva[3], Timofey Fedotenko[6,7], Konstantin Glazyrin[7], Dimitrios Bessas[8], Georgios Aprilis[8], Alexandr I. Chumakov[8], Hiroyuki Kagi[9], Leonid Dubrovinsky[3]

[1] – School of Biological, Earth and Environmental Sciences, University College Cork, Ireland

[2] – Institute of Precambrian Geology and Geochronology of the Russian Academy of Sciences, nab. Makarova 2, St. Petersburg 199034, Russia

[3] – Bayerisches Geoinstitut, Universität Bayreuth, D-95440 Bayreuth, Germany

[4] – A.N. Zavaritsky Institute of Geology and Geochemistry, 15 Vonsovskogo street, Ekaterinburg, 620016, Russia

[5] – Ural State Mining University, 30 Kuibysheva street, Ekaterinburg, 620014, Russia

[6] – Materials Physics and Technology at Extreme Conditions, Laboratory of Crystallography, Universität Bayreuth, D-95440 Bayreuth, Germany

[7] – Deutsches Elektronen-Synchrotron DESY, Notkestr. 85, 22607 Hamburg, Germany

[8] – ESRF-The European Synchrotron, CS 40220, 38043, Grenoble, Cedex 9, France

[9] – Geochemical Research Center, Graduate School of Science, The University of Tokyo, Tokyo 113-0033, Japan

*corresponding author, kate.kiseeva@ucc.ie

Contents

Contents 2
Supplementary Tables 3
Supplementary Table 1 3
Supplementary Table 2 4
Supplementary Table 3 5
Supplementary Table 4 6
Supplementary Table 5 10
Supplementary Figures 11
Supplementary Figure 1 11
Supplementary Figure 2 12
Supplementary Figure 3 13
Supplementary Figure 4 13
Supplementary Figure 5 14
References for Figure 1 14
References for Figure 3 15
References 15

Supplementary Tables

Supplementary Table 1. *Major and minor element concentrations in ferropericlase and magnesiowüstite, wt%*

Sample	SiO_2	TiO_2	Al_2O_3	Cr_2O_3	FeO	MnO	MgO	CaO	Na_2O	NiO	Total	Mg#
Magnesiowüstite												
SL82	0.11	-	-	0.06	86.81	1.68	9.35	0.04	-	0.06	98.11	16.1
SL5_2	0.04	-	-	1.25	70.4	0.69	26.16	b.d.l.	-	0.12	98.66	39.8
Ferropericlase												
SL14_2	0.04	b.d.l.	0.07	0.39	31.51	0.17	65.56	b.d.l.	0.04	0.96	98.74	78.8
SL14	0.06	0.03	0.08	0.28	27.49	0.19	69.84	-	0.05	1.15	99.17	81.9
SL24	0.06	-	-	0.53	24.28	0.29	74.29	b.d.l.	-	1.19	100.64	84.5

* b.d.l. – below detection limit

Supplementary Table 2. *Crystal structure data of inclusions (Fp - ferropericlase, Mfr – magnesioferrite, Mw - magnesiowüstite)*

**Inclusion**	**SL5_2**		**SL14**	**SL14_2**	**SL24**	**SL82**	
Phase	*Mw*	*Mfr*	*Fp*	*Fp*	*Fp*	*Mw*	*Mfr*
Chemical formula from XRD	$Fe_{0.556}Mg_{0.444}O$	$Fe_{2.183}Mg_{0.817}O_4$	$Fe_{0.112}Mg_{0.887}O$	$Fe_{0.225}Mg_{0.775}O$	$Fe_{0.151}Mg_{0.849}O$	$Fe_{0.817}Mg_{0.183}O$	$Fe_{2.383}Mg_{0.617}O_4$
Crystal system, space group	Cubic, $Fd\bar{3}m$	Cubic, $Fd\bar{3}m$	Cubic, $Fm\bar{3}m$	Cubic, $Fm\bar{3}m$	Cubic, $Fm\bar{3}m$	Cubic, $Fm\bar{3}m$	Cubic, $Fd\bar{3}m$
a (Å)	4.2853 (3)	8.3788 (4)	4.2266 (2)	4.2394 (2)	4.2308 (2)	4.2993 (3)	8.3695 (2)
V (Å ^{3})	78.69 (2)	588.23 (8)	75.51 (1)	76.19 (1)	75.73(1)	79.47 (2)	586.27 (4)
Radiation type	Synchrotron, λ= 0.2885 Å						
No. of measured, independent and observed [I>3σ(f)] reflections	88<br>23<br>23	625<br>116<br>101	105<br>21<br>21	90<br>26<br>26	110<br>28<br>28	56<br>22<br>21	534<br>120<br>97
R_{int}	0.026	0.047	0.016	0.034	0.023	0.041	0.019
θ values (°)	θ_{max} = 17.0<br>θ_{min} = 3.3	θ_{max} = 18.1<br>θ_{min} = 4.3	θ_{max} = 17.4<br>θ_{min} = 3.4	θ_{max} = 16.4<br>θ_{min} = 3.4	θ_{max} = 17.4<br>θ_{min} = 3.4	θ_{max} = 16.1<br>θ_{min} = 3.4	θ_{max} = 17.7<br>θ_{min} = 2.8
$(\sin\theta/\lambda)_{max}$ (Å $^{-1}$)	1.010	1.074	1.031	0.973	1.057	0.959	1.048
Range of h, k, l	*h* = −4→5<br>*k* = −7→6<br>*l* = −6→6	*h* = −13→14<br>*k* = −13→14<br>*l* = −11→10	*h* = −6→6<br>*k* = −5→6<br>*l* = −6→6	*h* = −8→8<br>*k* = −6→6<br>*l* = −5→5	*h* = −7→7<br>*k* = −3→5<br>*l* = −6→8	*h* = −6→7<br>*k* = −5→7<br>*l* = −5→5	*h* = −13→9<br>*k* = −13→13<br>*l* = −8→10
R [F^2>2 σ (F^2)], wR(F^2), S	0.020<br>0.043<br>1.16	0.033<br>0.080<br>1.13	0.019<br>0.044<br>1.40	0.021<br>0.044<br>1.07	0.016,<br>0.034,<br>1.07	0.019<br>0.056<br>1.33	0.012<br>0.025<br>0.98
No. of parameters	4	9	4	4	4	4	9

Supplementary Table 3. *Hyperfine parameters derived from room temperature Mössbauer spectra of the inclusions CS-centre shift (relative to α-Fe), QS-quadrupole splitting, FWHM-full width at half-maximum, B-hyperfine field, Fp - ferropericlase, Mfr – magnesioferrite, Mw - magnesiowüstite. The quadrupole shift of magnesioferrite sextets is zero within analytical uncertainty.*

Sample	Subspectra	CS, mm/s	QS, mm/s	FWHM, mm/s	B, T	I, %
SL14_2	Fe^{2+}_{Fp}	1.039(8)	0.69(1)	0.28(2)	-	100
SL24	Fe^{2+}_{Fp}	1.023(16)	0.67(3)	0.29(5)	-	100
SL14	Fe^{2+}_{Fp}	1.00(16)	0.62(3)	0.28(6)	-	100
SL5_2	Fe^{2+}_{Mw}	1.028(16)	1.66(11)	0.26(9)	-	15(6)
	Fe^{2+}_{Mw}	1.033(3)	0.79(2)	0.32(2)	-	85(6)
SL82	Fe^{2+}_{Mw}	1.04(2)	0.73(3)	0.46(7)	-	20(4)
	Fe^{3+}_{Mfr} (tetr/oct)	0.33(3)	~0	0.48(7)	48.1(2)	19(6)
	$Fe^{2.5+}_{Mfr\ (oct)}$	0.65(3)	~0	0.87(2)	44.7(3)	61(6)

Supplementary Table 4. *Measured and calculated chemical compositions of ferropericlase (Fp), magnesioferrite (Mfr) and magnesiowüstite (Mw), wt%*

N	Sample	Mineral	Approach	Fe^{3+}	Fe^{3+}/Fe_{tot}	SiO_2	TiO_2	Al_2O_3	Cr_2O_3
**1**	**SL82**	***Mw***	EPMA *(Supplementary Table 1)*	SMS	0.00	0.11	-	-	0.06
**2**	**SL82**	***Mw***	X-ray diffraction data *(Supplementary Table 2)*	SMS	0.00	-	-	-	-
**3**	**SL82**	***Mfr***	X-ray diffraction data *(Supplementary Table 2) Mg ↔ Fe^{3+} within tetrahedral position [crystal structure of defected inverse spinels]*	Calc	**0.58**	-	-	-	-
**4**	**SL82**	***Mfr***	X-ray diffraction and Mössbauer data *(Supplementary Tables 2 and 3) Mg ↔ Fe^{3+} within tetrahedral position [crystal structure of defected inverse spinels]*	SMS	**0.59**	-	-	-	-
**5**	**SL5_2**	***Mw***	EPMA *(Supplementary Table 1)*	SMS	0.00	0.04	-	-	1.25
**6**	**SL5_2**	***Mw***	X-ray diffraction data *(Supplementary Table 2)*	SMS	0.00	-	-	-	-
**7**	**SL5_2**	***Mfr***	X-ray diffraction data *(Supplementary Table 2) Mg ↔ Fe^{3+} within tetrahedral position [crystal structure of defected inverse spinels]*	Calc	**0.54**	-	-	-	-
**8**	**SL14_2**	***Fp***	EPMA *(Supplementary Table 1)*	SMS	0.00	0.04	b.d.l.	0.07	0.39
**9**	**SL14_2**	***Fp***	X-ray diffraction data *(Supplementary Table 2)*	SMS	0.00	-	-	-	-
**10**	**SL14**	***Fp***	EPMA *(Supplementary Table 1)*	SMS	0.00	0.06	0.03	0.08	0.28
**11**	**SL14**	***Fp***	X-ray diffraction data *(Supplementary Table 2)*	SMS	0.00	-	-	-	-
**12**	**SL24**	***Fp***	EPMA *(Supplementary Table 1)*	SMS	0.00	0.06	-	-	0.53
**13**	**SL24**	***Fp***	X-ray diffraction data *(Supplementary Table 2)*	SMS	0.00	-	-	-	-

Table 4 *(continued)*

Oxides (wt%)								Apfu									
Fe_2O_3	FeO	MnO	MgO	CaO	Na_2O	NiO	Total	Si	Ti	Al	Cr	Fe^{3+}	Fe^{2+}	Mn	Mg	Ca	Na
-	86.81	1.68	9.35	0.04	-	0.06	98.12	0.001	0.000	0.000	0.001	0.000	0.823	0.016	0.158	0.000	0.000
-	87.16	-	10.96	-	-	-	98.12	0.000	0.000	0.000	0.000	0.000	0.817	0.000	0.183	0.000	0.000
53.31	34.68	-	12.01	-	-	-	100.00	0.000	0.000	0.000	0.000	1.383	1.000	0.000	0.617	0.000	0.000
53.95	34.02	-	12.03	-	-	-	100.00	0.000	0.000	0.000	0.000	1.397	0.979	0.000	0.617	0.000	0.000
-	70.40	0.69	26.16	b.d.l.	-	0.12	98.66	0.000	0.000	0.000	0.010	0.000	0.590	0.006	0.390	0.000	0.000
-	68.13	-	30.53	-	-	-	98.66	0.000	0.000	0.000	0.000	0.000	0.556	0.000	0.444	0.000	0.000
47.41	36.06	-	16.53	-	-	-	100.00	0.000	0.000	0.000	0.000	1.183	1.000	0.000	0.817	0.000	0.000
-	31.51	0.17	65.56	b.d.l.	0.04	0.96	98.74	0.000	0.000	0.001	0.002	0.000	0.210	0.001	0.778	0.000	0.001
-	33.67	-	65.07	-	-	-	98.74	0.000	0.000	0.000	0.000	0.000	0.225	0.000	0.775	0.000	0.000
-	27.49	0.19	69.84	-	0.05	1.15	99.14	0.000	0.000	0.001	0.002	0.000	0.178	0.001	0.809	0.000	0.001
-	18.34	-	80.80	-	-	-	99.14	0.000	0.000	0.000	0.000	0.000	0.113	0.000	0.887	0.000	0.000
-	24.28	0.29	74.29	b.d.l.	-	1.19	100.64	0.000	0.000	0.000	0.003	0.000	0.153	0.002	0.833	0.000	0.000
-	18.62	-	82.02	-	-	-	100.64	0.000	0.000	0.000	0.000	0.000	0.113	0.000	0.887	0.000	0.000

* b.d.l. – below detection limit

Table 4 *(continued)*

Ni	∑ (cat.)	∑ (ch.)	Chemical Formula (0.0x apfu)		Chemical Formula (0.00x apfu)
0.001	1.000	2.003	$Fe_{0.82} Mg_{0.16}Mn_{0.02}O$	or	$Fe_{0.823} Mg_{0.158}Mn_{0.016}Si_{0.001}Cr_{0.001}Ni_{0.001} O$
0.000	1.000	2.000	$Fe_{0.82} Mg_{0.18} O$	or	$Fe_{0.817} Mg_{0.183} O$
0.000	3.000	7.383	$Tet(Fe^{3+}_{0.38} Mg_{0.62})_{1.00} Oct(Fe^{3+}_{1.00} Fe^{2+}_{1.00})_{2.00} O_4$		$Tet(Fe^{3+}_{0.383} Mg_{0.617})_{1.000} Oct(Fe^{3+}_{1.000} Fe^{2+}_{1.000})_{2.000} O_4$
0.000	2.993	7.383	$Tet(Fe^{3+}_{0.38} Mg_{0.62})_{1.00} Oct(Fe^{3+}_{1.01} Fe^{2+}_{0.98})_{1.99} O_4$	or	$Tet(Fe^{3+}_{0.383} Mg_{0.617})_{1.000} Oct(Fe^{3+}_{1.014} Fe^{2+}_{0.979})_{1.993} O_4$
0.001	0.997	2.005	$Fe_{0.59} Mg_{0.39}Mn_{0.01}Cr_{0.01} O$	or	$Fe_{0.590} Mg_{0.390}Mn_{0.006}Cr_{0.010}Ni_{0.001} O$
0.000	1.000	2.000	$Fe_{0.56} Mg_{0.44} O$	or	$Fe_{0.556} Mg_{0.444} O$
0.000	3.000	7.183	$Tet(Fe^{3+}_{0.18} Mg_{0.82})_{1.00} Oct(Fe^{3+}_{1.00} Fe^{2+}_{1.00})_{2.00} O_4$	or	$Tet(Fe^{3+}_{0.183} Mg_{0.817})_{1.000} Oct(Fe^{3+}_{1.000} Fe^{2+}_{1.000})_{2.000} O_4$
0.006	0.999	2.000	$Mg_{0.78} Fe_{0.21}Ni_{0.01} O$		$Mg_{0.778} Fe_{0.210}Ni_{0.006}Cr_{0.002}Al_{0.001}Mn_{0.001}Na_{0.001} O$
0.000	1.000	2.000	$Mg_{0.78} Fe_{0.22} O$	or	$Mg_{0.775} Fe_{0.225} O$
0.007	0.999	2.000	$Mg_{0.81} Fe_{0.18}Ni_{0.01} O$		$Mg_{0.809} Fe_{0.178}Ni_{0.007}Cr_{0.002}Al_{0.001}Mn_{0.001}Na_{0.001} O$
0.000	1.000	2.000	$Mg_{0.89} Fe_{0.11} O$	or	$Mg_{0.887} Fe_{0.113} O$
0.007	0.998	1.999	$Mg_{0.83} Fe_{0.15}Ni_{0.01} O$		$Mg_{0.833} Fe_{0.153}Ni_{0.007}Cr_{0.003}Mn_{0.002} O$
0.000	1.000	2.000	$Mg_{0.89} Fe_{0.11} O$	or	$Mg_{0.887} Fe_{0.113} O$

Fp	- Ferropericlase
Mw	- Magnesiowüstite
Mfr	- Magnesioferrite
∑ (cat.)	- Total of the cation sum
∑ (ch.)	- Charge balance

N1, 5, 8, 10 and 12

Apfu composition and structural formulae of ferropericlases have been calculated directly from the EPMA data (Supplementary Table 1). The obtained total of cation numbers [∑ (cat.)] and charge balance [∑ (ch.)] showed that the ferropericlases do not contain Fe_2O_3 (or its concentration is negligible).

N2, 6, 9, 11 and 13

Ferropericlase composition (in oxide wt%) has been calculated based on the X-ray diffraction data (Supplementary Table 2). Totals (wt%) for these compositions are the same as for the EPMA analyses to ensure comparability of the data. Fe_2O_3 (wt%), Fe^{3+} and Fe^{2+} have been calculated from the total of cation numbers [∑ (cat.)] and charge balance [∑ (ch.)].

N3 and 7

Magnesioferrite composition (in oxide wt%) has been calculated based on the X-ray diffraction data (Supplementary Table 2). Totals for these compositions were taken as 100 wt%. Fe_2O_3 (wt%), Fe^{3+} and Fe^{2+} have been calculated from the total of cation numbers [∑ (cat.)] and charge balance [∑ (ch.)].

N4

Magnesioferrite composition (in oxide wt%) has been calculated based on the X-ray diffraction data (Supplementary Table 2). Totals for these compositions were taken as 100 wt%. Fe_2O_3 (wt%), Fe^{3+} and Fe^{2+} have been calculated from the Mössbauer data (Supplementary Table 3).

Supplementary Table 5. *Physical and chemical characteristics of diamonds enclosing the studied inclusions (from ref. 1).*

Sample	Type	N, ppm, max	N, ppm, min	$\delta^{13}C$, ‰, max	$\delta^{13}C$, ‰, min
SL5_2	IaB	25	15	-6.0	-2.5
SL14_2	IIa	0.01	0.07	-4.5	-2.5
SL24	IaB	14	14	-4.5	-3.7
SL82	IIa	-	-	-5.2	-4.2
SL14	IaB	192	122	-5.6	-5.2

Supplementary Figures

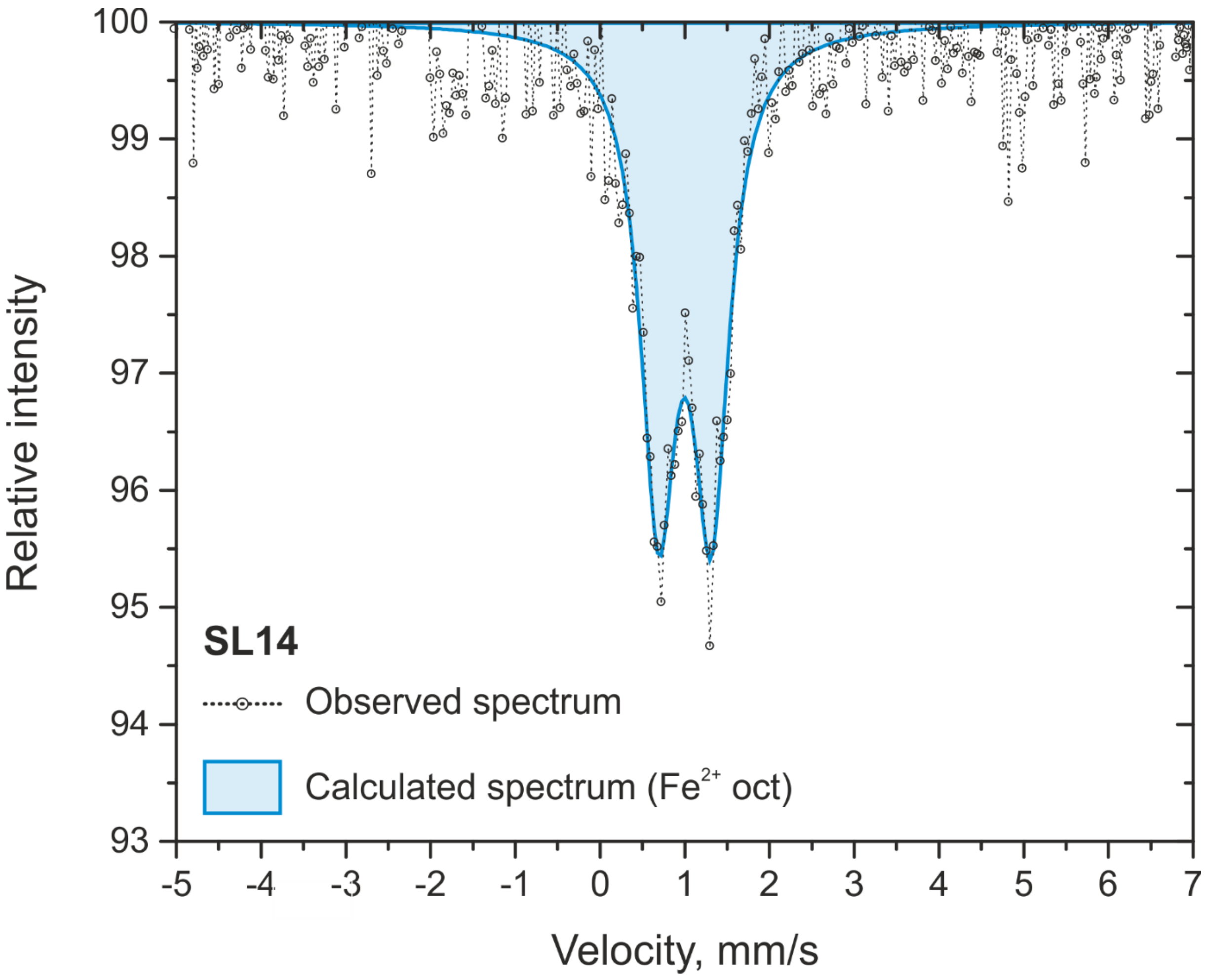

Supplementary figure 1. *Mössbauer spectrum of inclusion SL14*

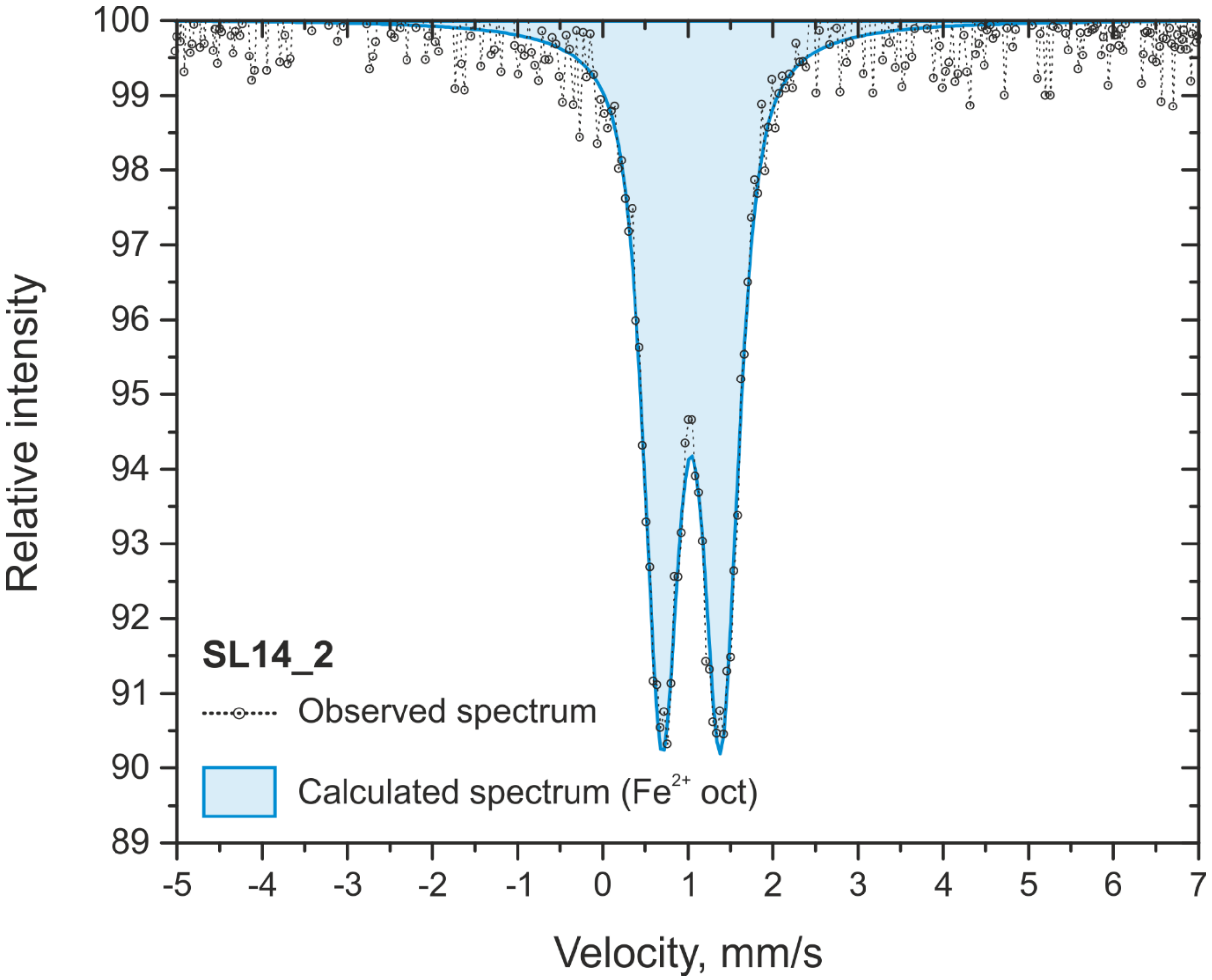

Supplementary figure 2. *Mössbauer spectrum of inclusion SL14_2.*

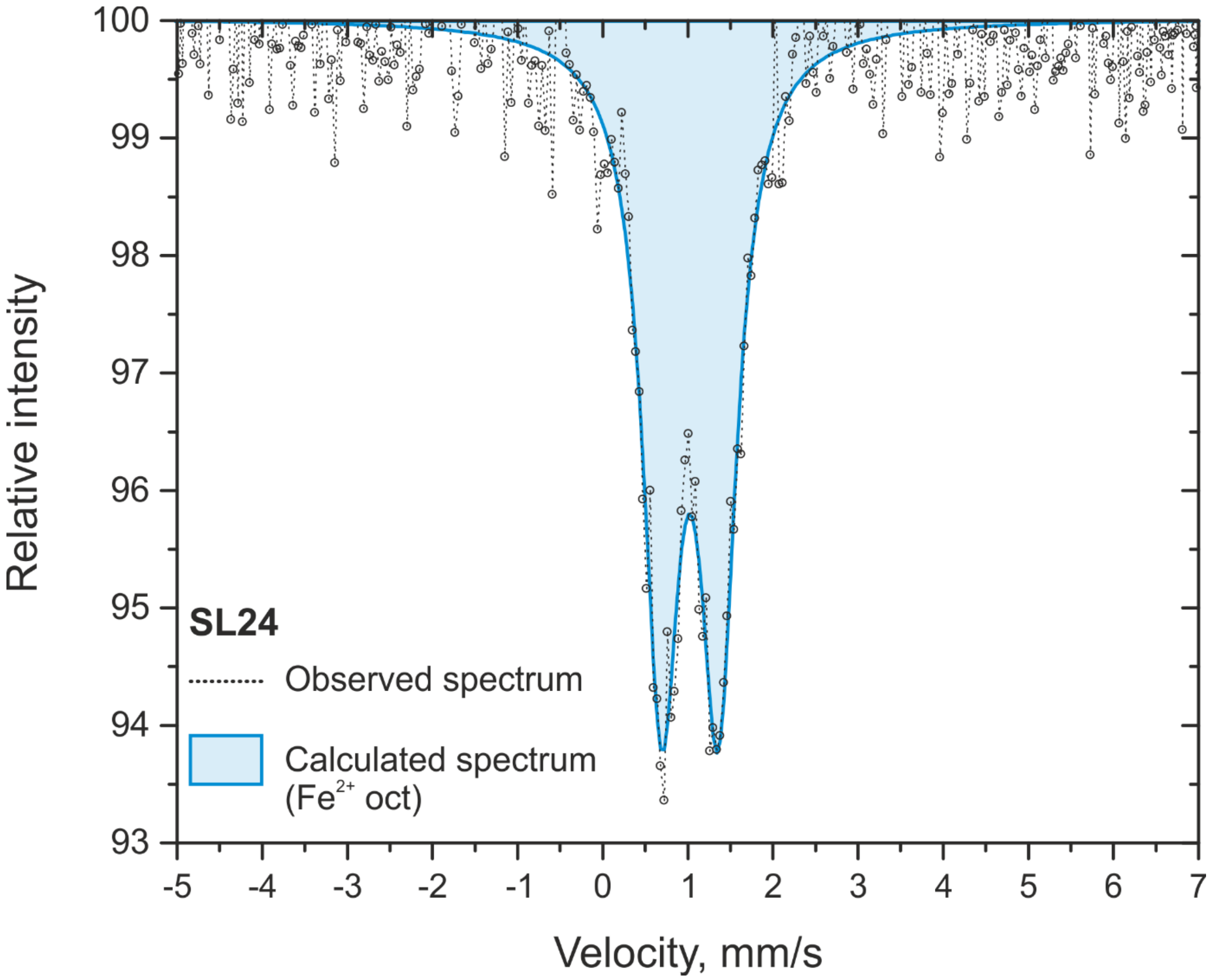

Supplementary figure 3. *Mössbauer spectrum of inclusion SL24.*

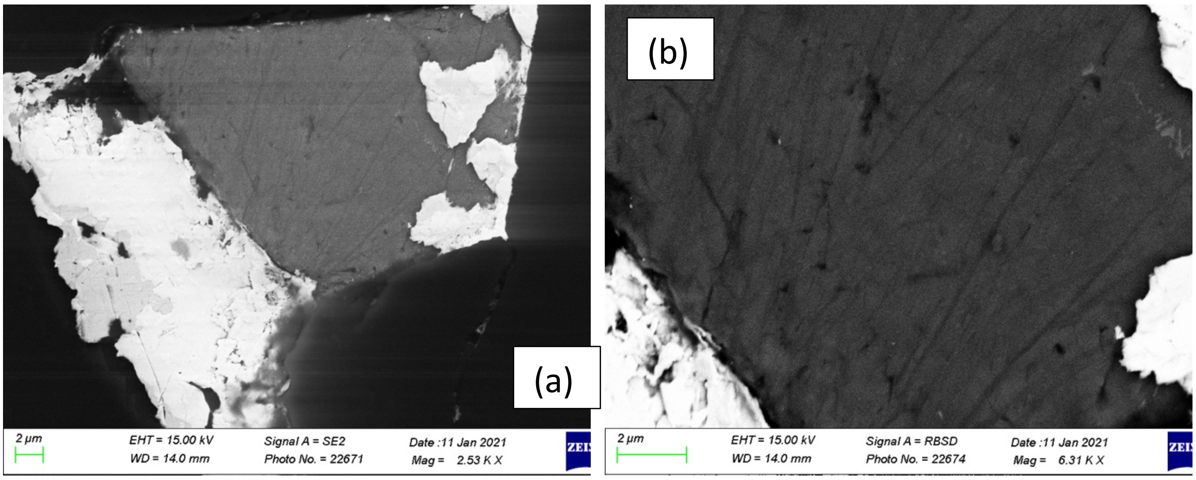

Supplementary figure 4. *Back-scattered electron image of inclusion SL82 showing no visible exsolution of magnesioferrite. (a) entire inclusion, with gold coating at the edge and on top. (b) enlargement of the central area.*

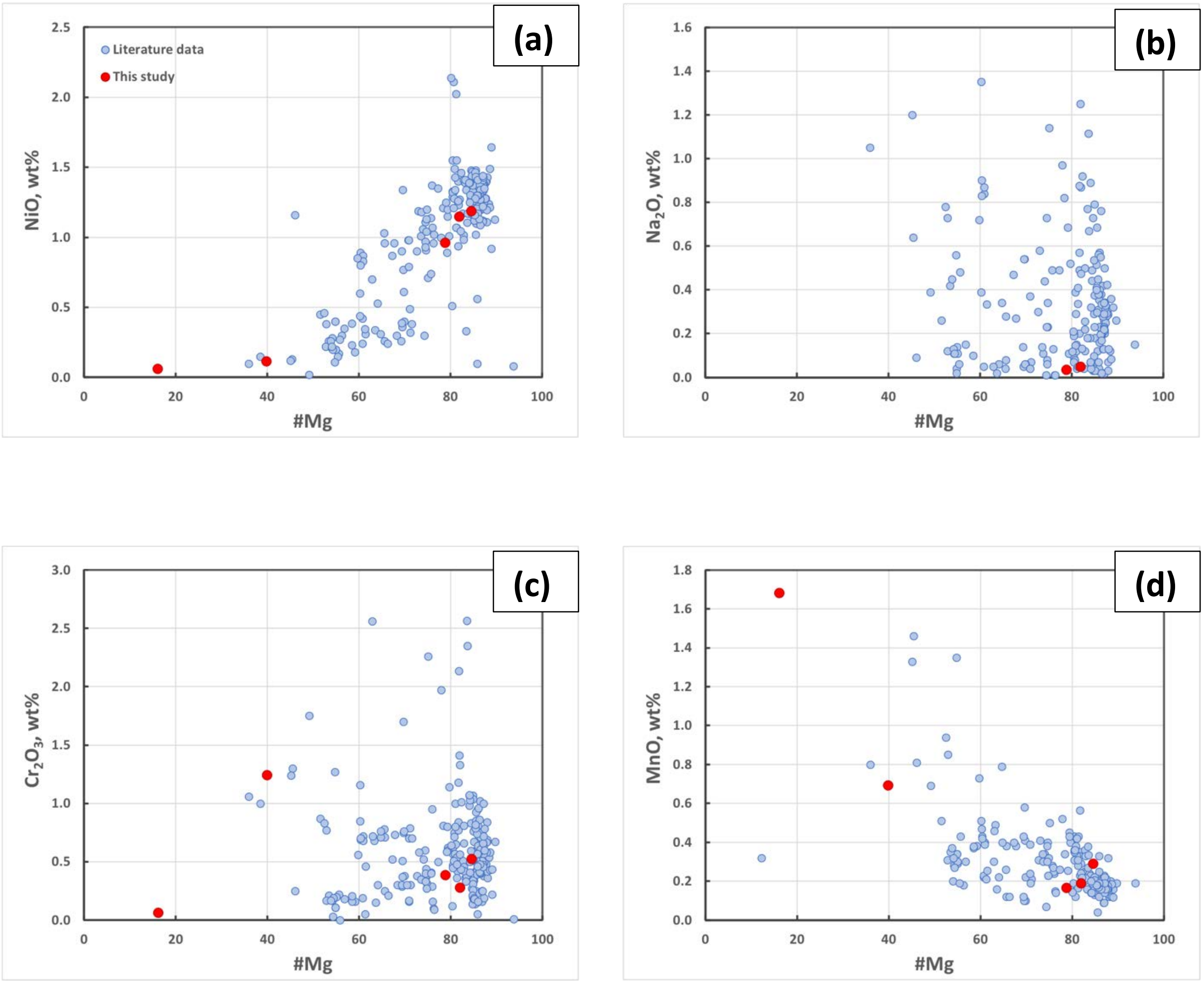

Supplementary figure 5. *Concentrations of NiO (a), Na_2O (b), Cr_2O_3 (c) and MnO (d) in ferropericlase-magnesioferrite inclusions reported in the literature.*

References for Figure 1

Following references were used for compiling Figure 1.

Experimental data for Figure 1 was sourced from references 2–7. Data on natural ferropericlase and magnesiowüstite compositions were sourced from references 1, 7–33 and where inaccessible, literature data compiled by Thomson et al. (ref. 34).

References for Figure 3

Data for compiling this figure were sourced from references 1, 10, 11, 13, 14, 18–22, 25–29, and 35.

* The mineral symbols used here and in the main article are according to Warr (ref. 36).

References

1 Zedgenizov, D. A., Kagi, H., Shatsky, V. S. & Ragozin, A. L. Local variations of carbon isotope composition in diamonds from Sao-Luis (Brazil): Evidence for heterogenous carbon reservoir in sublithospheric mantle. *Chemical Geology* **363**, 114-124 (2014).

2 Ishii, T., Kojitani, H. & Akaogi, M. Post-spinel transitions in pyrolite and Mg_2SiO_4 and akimotoite–perovskite transition in $MgSiO_3$: Precise comparison by high-pressure high-temperature experiments with multi-sample cell technique. *Earth and Planetary Science Letters* **309**, 185-197 (2011).

3 Kesson, S., Gerald, J. F. & Shelley, J. Mineralogy and dynamics of a pyrolite lower mantle. *Nature* **393**, 252-255 (1998).

4 Herzberg, C. & Zhang, J. Z. Melting experiments on anhydrous peridotite KLB-1: Compositions of magmas in the upper mantle and transition zone. *Journal of Geophysical Research-Solid Earth* **101**, 8271-8295 (1996).

5 Hirose, K. Phase transitions in pyrolitic mantle around 670-km depth: Implications for upwelling of plumes from the lower mantle. *Journal of Geophysical Research-Solid Earth* **107** (2002).

6 Nishiyama, N. & Yagi, T. Phase relation and mineral chemistry in pyrolite to 2200 degrees C under the lower mantle pressures and implications for dynamics of mantle plumes. *Journal of Geophysical Research-Solid Earth* **108** (2003).

7 Walter, M., Nakamura, E., Trønnes, R. & Frost, D. Experimental constraints on crystallization differentiation in a deep magma ocean. *Geochimica et Cosmochimica Acta* **68**, 4267-4284 (2004).

8 Anzolini, C. *et al.* Evidence for complex iron oxides in the deep mantle from FeNi(Cu) inclusions in superdeep diamond. *Proceedings of the National Academy of Sciences* **117**, 21088-21094 (2020).

9 Cid, J. P., Nardi, L., Cid, C. P., Gisbert, P. E. & Balzaretti, N. Acid compositions in a veined-lower mantle, as indicated by inclusions of (K, Na)-hollandite+ SiO2 in diamonds. *Lithos* **196**, 42-53 (2014).

10 Donnelly, C. L., Stachel, T., Creighton, S., Muehlenbachs, K. & Whiteford, S. Diamonds and their mineral inclusions from the A154 South pipe, Diavik Diamond Mine, Northwest territories, Canada. *Lithos* **98**, 160-176 (2007).

11 Harte, B., Harris, J. W., Hutchison, M. T., Watt, G. R. & Wilding, M. C. Lower mantle mineral associations in diamonds from Sao Luiz, Brazil. *Mantle petrology: Field observations and high-pressure experimentation: A tribute to Francis R.(Joe) Boyd* **6**, 125-153 (1999).

12 Hayman, P. C., Kopylova, M. G. & Kaminsky, F. V. Lower mantle diamonds from Rio Soriso (Juina area, Mato Grosso, Brazil). *Contributions to Mineralogy and Petrology* **149**, 430-445 (2005).

13 Kaminsky, F. V., Khachatryan, G. K., Andreazza, P., Araujo, D. & Griffin, W. L. Super-deep diamonds from kimberlites in the Juina area, Mato Grosso State, Brazil. *Lithos* **112**, 833-842, (2009).

14 McDade, P. & Harris, J. in *International Kimberlite Conference: Extended Abstracts.* 561-563, (1999).

15 Otter, M. L. Diamonds and their mineral inclusions from the Sloan diatremes of the Colorado-Wyoming State Line kimberlite district, North America. PhD thesis, University of Cape Town (1989).

16 Shatsky, V., Zedgenizov, D., Ragozin, A. & Kalinina, V. Diamondiferous subcontinental lithospheric mantle of the northeastern Siberian Craton: Evidence from mineral inclusions in alluvial diamonds. *Gondwana Research* **28**, 106-120 (2015).

17 Sobolev, N., Yefimova, E. & Koptil, V. Crystalline inclusions in diamonds in the northeast of the Yakutian diamondiferous province. *In International Kimberlite Conference: Extended Abstracts.* 832-834 (1998).

18 Stachel, T., Harris, J. W., Aulbach, S. & Deines, P. Kankan diamonds (Guinea) III: $\delta^{13}C$ and nitrogen characteristics of deep diamonds. *Contributions to Mineralogy and Petrology* **142**, 465-475 (2002).

19 Stachel, T., Harris, J. W. & Brey, G. P. Rare and unusual mineral inclusions in diamonds from Mwadui, Tanzania. *Contributions to Mineralogy and Petrology* **132**, 34-47 (1998).

20 Hutchison, M. T. *Constitution of the deep transition zone and lower mantle shown by diamonds and their inclusions* Unpubl. PhD thesis, University of Edinburgh (1997).

21 Kaminsky, F. V. *et al.* Superdeep diamonds from the Juina area, Mato Grosso State, Brazil. *Contributions to Mineralogy and Petrology* **140**, 734-753, (2001).

22 Tappert, R., Stachel, T., Harris, J. W., Shimizu, N. & Brey, G. P. Mineral inclusions in diamonds from the Panda kimberlite, Slave Province, Canada. *European Journal of Mineralogy* **17**, 423-440 (2005).

23 Kopylova, M. G., Gurney, J. J. & Daniels, L. R. M. Mineral inclusions in diamonds from the River Ranch kimberlite, Zimbabwe. *Contributions to Mineralogy and Petrology* **129**, 366-384 (1997).

24 Moore, R., Otter, M., Rickard, R., Harris, J. & Gurney, J. The occurrence of moissanite and ferro-periclase as inclusions in diamond. *In International Kimberlite Conference: Extended Abstracts.* 409-411 (1986).

25 Stachel, T., Harris, J. W., Brey, G. P. & Joswig, W. Kankan diamonds (Guinea) II: lower mantle inclusion parageneses. *Contributions to Mineralogy and Petrology* **140**, 16-27 (2000).

26 Wilding, M. C. *A study of diamonds with syngenetic inclusions* PhD thesis, University of Edinburgh (1990).

27 Tappert, R. *et al.* Deep mantle diamonds from South Australia: A record of Pacific subduction at the Gondwanan margin. *Geology* **37**, 43-46 (2009).

28 Burnham, A. D. *et al.* Diamonds from the Machado River alluvial deposit, Rondonia, Brazil, derived from both lithospheric and sublithospheric mantle. *Lithos* **265**, 199-213 (2016).

29 Davies, R. A., Griffin, W. L., O'Reilly, S. Y. & Doyle, B. J. Mineral inclusions and geochemical characteristics of microdiamonds from the DO27, A154, A21, A418, DO18, DD17 and Ranch Lake kimberlites at Lac de Gras, Slave Craton, Canada. *Lithos* **77**, 39-55 (2004).

30 Davies, R. A., Griffin, W. L., O'Reilly, S. Y. & McCandless, T. E. Inclusions in diamonds from the K14 and K10 kimberlites, Buffalo Hills, Alberta, Canada: diamond growth in a plume? *Lithos* **77**, 99-111 (2004).

31 Zedgenizov, D., Yefimova, E., Logvinova, A., Shatsky, V. & Sobolev, N. Ferropericlase inclusions in a diamond microcrystal from the Udachnaya kimberlite pipe, Yakutia. Doklady Earth Sciences 319-321 (2001).

32 Scott-Smith, B., Danchin, R., Harris, J. & Stracke, K. in *Developments in Petrology* **11**, 121-142 (1984).

33 Van Rythoven, A. & Schulze, D. In-situ analysis of diamonds and their inclusions from the Diavik Mine, Northwest Territories, Canada: Mapping diamond growth. *Lithos* **112**, 870-879 (2009).

34 Thomson, A. R., Walter, M. J., Kohn, S. C. & Brooker, R. A. Slab melting as a barrier to deep carbon subduction. *Nature* **529**, 76-79 (2016).

35 Griffin, W. L. *et al.* Layered mantle lithosphere in the Lac de Gras area, Slave Craton: Composition, structure and origin. *Journal of Petrology* **40**, 705-727 (1999).

36 Warr, L. N. IMA–CNMNC approved mineral symbols. *Mineralogical Magazine* **85**(3), 291-320 (2021).
